# Left branch of portal vein thrombosis in a liver transplant recipient with donation after cardiac death donor: A case report

**DOI:** 10.1097/MD.0000000000005520

**Published:** 2016-12-09

**Authors:** Maogen Chen, Weiqiang Ju, Xiaohong Lin, Qiang Zhao, Dongping Wang, Xiaoshun He

**Affiliations:** a Organ Transplant Center, The First Affiliated Hospital, Sun Yat-sen University; b Guangdong Provincial Key Laboratory of Organ Donation and Transplant Immunology, Guangdong Provincial International Cooperation Base of Science and Technology (Organ Transplantation); c Department of Thyroid and Breast Surgery, the First Affiliated Hospital of Sun Yat-sen University, 58 Zhongshan Er Road, 510080 Guangzhou, China.

**Keywords:** liver transplantation, portal vein thrombosis, thrombolytic therapy

## Abstract

**Introduction:**

Portal vein thrombosis (PVT) is one of the most severe complications after liver transplantation, which usually causes graft loss and recipient mortality. The founding of the embolic branch of portal system and its result are not well described in the literature.

**Clinical findings and diagnoses:**

We report here an unusual case of complete right branch thrombosis of portal vein after orthotopic liver transplantation from a donation after cardiac death donor, without obvious malaise.

**The interventions and outcomes:**

The branch thrombosis of portal vein was detected by Doppler ultrasound 11 days after transplantation, followed by angiography for further verification. After treatment with urgent indwelling catheter thrombolysis with urokinase, results improved and the patient showed stable liver function. The patient has been in remission for 22 months with normal graft function.

**Conclusion:**

In this case report, we show that frequent ultrasound inspection should be adopted to detect the unobstructed vessel in the early stage of liver transplantation, and local thrombolytic therapy can be used to prevent liver injury and keep the vessel open.

## 1 Introduction

Although the technical progress had been made in recent dacades, vascular complications in liver transplantation are still one of major element that results in allograft loss, increases postoperative morbidity and mortality. Vascular complications include arterial complications and venous complications occurred in up to 9% of patients.^[1]^ Arterial complications are common in the early stage posttransplantation, which caused much of graft loss and recipient mortality. Venous complications are normally found in late postoperative recovery stage and are easier to treat.^[2]^ The most common venous complications is portal vein thrombosis (PVT), which occurs frequently in living donor liver transplantation.^[3]^ PVT was gradually developed by portal vein stenosis. The incidence of portal vein stenosis in pediatric living donor liver transplantation was about 8.2%.^[4]^

The treatment of PVT was easy but the key point was to detect earlier. Since there were no much symptoms in the early stage of PVT. LT recipients with late PVT usually have lower white blood cell, platelet counts, increased international normalized ratio and more gastrointestinal bleeding.^[5]^ The patient and allograft survival rates were 78% and 80% for PVT cases at 10 years, respectively.^[5]^ Following the application of ultrasonic examination technology, more and more PVT cases were discovered in the early stage of liver transplantation.

Here, we report a patient of PVT with complete obstruction of right branch of intrahepatic porta vein on day 11 postliver transplantation. The patient was cured by locally continuous thrombolytic therapy using ultrasonic and intervenient methods. Written informed consent was obtained from the patient for the publication of this case report and any accompanying images. The research protocol was approved by the Human Research Ethics Committee in the First Affiliated Hospital of Sun Yat-sen University.

## 2 Case report

The patient was male, 56 years old. He was diagnosed as primary liver carcinoma complicated with a 20 years history of HBV-related liver cirrhosis, 3 years of hypertension, and 2 years of type II diabetes. He suffered with 3 times of transcatheter hepatic arterial chemoembolization, 1 time of percutaneous transhepatic sonographically guided radiofrequency ablation of liver cancer, and 1 time of laparoscopic microwave coagulation in Couinaud segment I due to uncontrolled liver carcinoma. The AFP was 36.13 μg/L. HBV DNA was lower that 100 IU/mL. Finally, he had successful LT from a donation after cardiac death donor. The preoperative model for end-stage liver disease score was 7. The operative course was uneventful.

The patient received tacrolimus-based immunosuppression with the serum level of 8 to 12 μg/L. Basiliximab (20 mg, Simulect) was used intravenously on day 0, 4 postoperative respectively as an immune induction therapy. Entecavir and hepatitis B immunoglobulin were used regularly to prevent hepatitis B recurrence. As the vascular anastomosis was satisfying, initial anticoagulation was not in regular usage postoperation.

On day 8 postoperation, ALT and AST, the indicator of graft function, were getting almostly normal. He suffered with hypoproteinemia that needed 2 to 3 doses of albumin to keep ALB at normal range. But the patient started to complain of edema in both lower limbs and scrotum. Draining fluid from abdominal cavity was getting more than before. Both collateral arteries and venous of lower extremity were subjected to ultrasonic examination by Doppler method, which showed as patent blood flow without embolism. However, Doppler ultrasound of the liver graft presented as thrombosis in the right portal vein and nubilous posthepatic inferior vena cava (IVC) on day 11 after transplantation. Contrast-enhanced ultrasound was further adopted to confirm the thrombosis with a size of 4.5 cm × 1.5 cm in the right portal vein. The liver graft was getting larger and the oblique diameter of right lobe of liver was 18.5 cm. The peak velocity right hepatic artery was 172.5 cm/s, resistent index (RI) 0.54, while the peak velocity left hepatic artery was 62.4 cm/s, RI 0.66.

**Figure 1:**
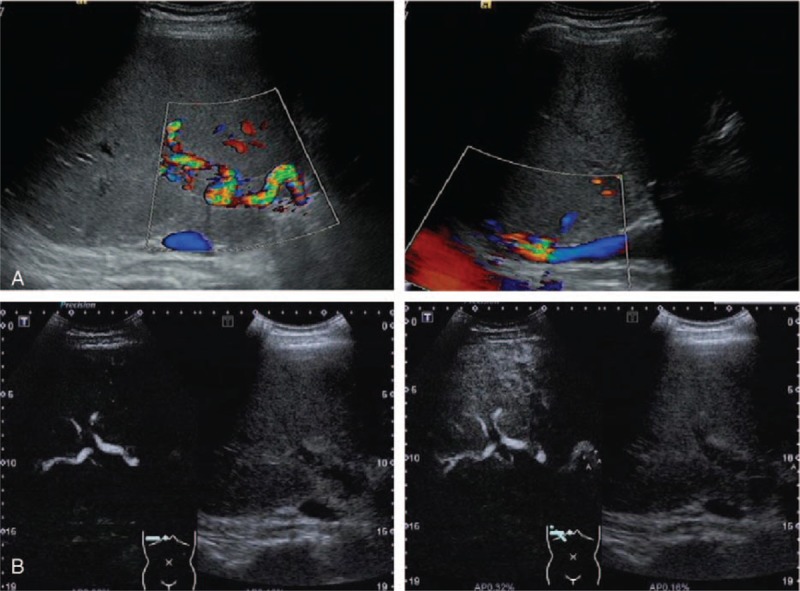
Doppler sonography was performed before initiation of thrombolysis. (A) Color Doppler ultrasonography showed no blood perfusion of the right branch of portal vein (left panel), as well as patent outflow vein of liver and inferior vena cava (right panel). (B) Real-time contrast-enhanced ultrasonography showed continuously lacking of blood flow in the right branch of portal vein, both in the portal and late phases.

Urgent angiography under local anesthesia was performed to identify the blood flow of IVC. Transcutaneous transfemoral catheterization portography revealed no obstruction of IVC and hepatic veins trunks. At the same time, we were trying to get rid of thrombosis in the right hepatic portal vein. We then decided to treat with indwelling catheter thrombolysis as the first therapeutic option. Since there was no dilated bile duct in the graft, it was difficult to guide therapeutic percutaneous puncture catheter to portal vein through interventional method. Therefore, we chose the ultrasound-guided transcutaneous transhepatic puncture to place a hydrophilic 0.035-inch guidewire (Terumo, Tokyo, Japan) to the portal trunk. Then, a 5-Fr sheath introducer (Medikit) was placed through the guidewire with tip close to the superior mesenteric vein under direct vision of the interventional therapy. Contrast agent was injected from the sheath introducer to the portal vein, and a lateral view of the DSA confirmed complete occlusion of the right portal trunk with development of varicose veins in the portasystemic collateral vessels (Fig. 1).

Infusion catheter (4-Fr Fountain Infusion System; Merit Medical OEM) was introduced into the portal trunk through percutaneous transhepatic tract (Fig. 1). Thrombolytic therapy was initiated with continuous infusion of urokinase (10000 U/kg/day) and low molecular weight heparin (4000 U/day). Closed observation was maintained regarding bleeding at the site of puncture and the entire body, especially gingival hemorrhage.

A Doppler sonography performed 3 times per week after initiation of thrombolytic therapy demonstrated gradually reduced embolism of right portal trunk. Contrast-enhanced ultrasound was used to recheck the state of thrombosis in the right portal vein after 3 days of thrombolysis therapy. The thrombosis shrinked at a size of 0.7 cm × 0.6 cm. Although the oblique diameter of right lobe of liver was the same size, the peak velocity right hepatic artery was getting lower as 79.7 cm/s. Two weeks later, ultrasound examination showed that the oblique diameter of right lobe of liver was 16.6 cm. There was no thrombosis in PV and patent blood flow of hepatic artery with a peak velocity of 56 cm/s, RI 0.65.

**Figure 2:**
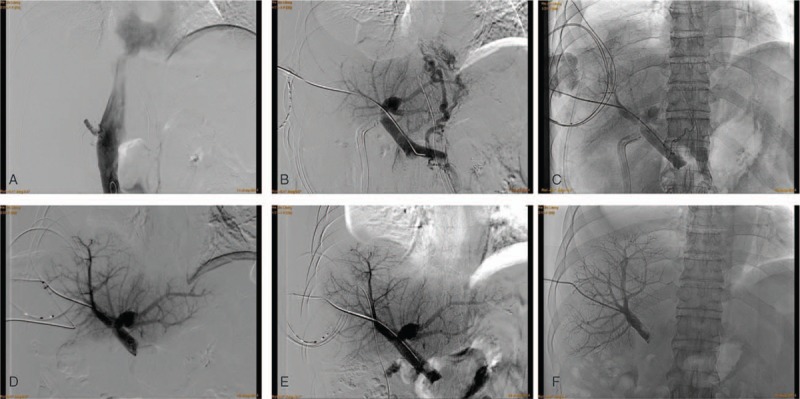
A lateral view of follow-up digital subtraction angiography (DSA) portography before or after the recanalization therapy. (A) Transfemoral catheterization of femoral vein portography was performed, which showed patent RHIVC. (B) Percutaneous transhepatic contrast injection of the portal vein before thrombolysis revealed persistent complete thrombosis of right branch of portal vein with retrograde filling of large gastric varices. As the thrombolysis therapy was ongoing, interventional portography showed gradually patent right branch of portal vein after 4 (C), 8 (D), 11 (E), and 15 (F) days after thrombolysis treatment. RHIVC = retrohepatic inferior vena cava.

A follow-up DSA with contrast injection from the infusion catheter 2 time per week after initiation of thrombolysis procedure revealed gradually patent portal vein trunk (Fig. 2). The catheter was removed after 2 weeks of thrombolytic therapy, and vascular blockage was proceeded for hemostasis.

After treatment with above-described interventional radiology methods, the patient discharged after recovery with anticoagulation therapy with bayaspirin (100 mg/day) for a long-term follow-up. Until this paper submission, the patient has been followed with normal graft function and patent portal vein for 22 months. Informed consent was obtained from the patient prior to treatment.

## 3 Discussion

In orthotopic full liver transplantation, the incidence of PVT was about 4% to 12.6% postliver transplantation.^[6–8]^ Patients with PVT had worse allograft survival and patient survival, longer length of stay and higher retransplantation rate than ones without PVT.^[8]^ Hibi et al^[6]^ summarized 174 cases of adult liver transplantation patients with PVT and reported that there were comparable survival rates between PVT group and no PVT group, so do both complete and partial PVT.

The fragile hemostatic balance might be a reason for the development of PVT, in that both prohemostatic and antihemostatic factors were affected when the transplanted liver suffered with ischemia reperfusion injury especially in donation after cardiac death derived organ. It is important to keep balance between hypocoagulable and hypercoagulable situation, which caused either hemorrhagic or thrombotic complications.^[8]^ In LT recipients, it has been reported that the occurrence rate of PVT was more in patients with HCC, autoimmune chronic active hepatitis, cryptogenic cirrhosis, alcoholic liver disease, or patients with previous treatment of portal hypertension-related bleeding.^[8]^ Orlandini et al^[9]^ found that the following factors, including portal vein diameter ≤3 mm, donor-to-recipient body weight ratio, prolonged ischemic time, and use of arterial grafts, associated with vascular complications in patients undergoing orthotopic liver transplantation. The incidence of vascular complications in patients with portal vein (PV) diameter >3 mm or ≤3 mm are about 20% or 50%, respectively, after 10 years of orthotopic liver transplantation.^[9]^ Although the survival rates between patients with PVT and patients without PVT had no difference, Neto et al^[3]^ proved that the use of vascular grafts in living donor liver transplantation was an independent risk factor for the occurrence of PVT. The other common factors include excessive length of portal vein, hypercoagulability, thrombus history, portal manipulation, and caliber discrepancies between the donor and recipient veins.^[1]^

For the treatment of PVT, the severity of thrombosis had no correlation to the outcome due to the effective management.^[10]^ Different surgical approaches could be employed to reconstruct the portal venous flow depending on the extent of thrombosis, and the availability of collateral vessels.^[8]^ Those treatment methods include endovascular thrombectomy alone, additional PV plasty, PV stenting, interposition graft, or additional interruption of collaterals. For those extensive PVT patients, complete PV thrombectomy was inefficacious especially at intrapancreatic embolus.^[2]^ Although for those recipients with portomesenteric venous thrombosis after liver transplantation, the consequences are mostly graft loss and retransplantation. Lorenz et al^[11]^ reported a case of liver transplant recipient that using the Trellis pharmacomechanical thrombolysis device could salvage the complete intra- and extra-hepatic portomesenteric thrombosis. When patients with PVT after liver transplantation occur at the extrahepatic portal vein, good alternative can be direct portal revascularization with the Meso-Rex bypass.^[12]^ When the PVT occurred slowly, we will observe subsequently portal flow collateral circulation through preexisting sizable small vessels or capillary vessels. However, when the patients complicated with portal hypertension, the porto-variceal anastomosis was useful to treat extensive porto-mesenteric thrombosis.^[13]^ For those patients with previously existed nontumorous PVT, interruption of sizable collaterals and intraoperative cine-portogram (IOP) were helpful for diagnosis and treatment of PVT and varicose collaterals.^[14]^ So in the PVT complication treatment choice, it may be a good choice using IOP in the process of retransplantion to prevent further rethrombosis.

## 4 Conclusion

As we reported here, PVT after LT could be cured by lots of strategies. But the key point was to find thrombosis early in PV with the help of Doppler ultrasound or CT examination. The underlying mechanism and etiology of PVT remain unclear, and there is hard to predict the occurrence of PVT in those patients with normal graft function.

## Acknowledgements

The authors thank the National Natural Science Foundation of China (81373156, 81401324, 81570587); Guangdong Provincial Key Laboratory of Organ Donation and Transplant Immunology (2013A061401007); and Guangdong Provincial International Cooperation Base of Science and Technology (Organ Transplantation) (2015B050501002) for the support.
